# Crystal structure and Hirshfeld surface analysis of 5,7-diphenyl-1,2,3,5,6,7-hexa­hydro­imidazo[1,2-*a*]pyridine-6,6,8-tricarbo­nitrile methanol mono­solvate

**DOI:** 10.1107/S2056989021004655

**Published:** 2021-05-11

**Authors:** Farid N. Naghiyev, Gunay Z. Mammadova, Ali N. Khalilov, Zeliha Atioğlu, Mehmet Akkurt, Anzurat A. Akobirshoeva, İbrahim G. Mamedov

**Affiliations:** a Department of Chemistry, Baku State University, Z. Khalilov str. 23, Az, 1148 Baku, Azerbaijan; b "Composite Materials" Scientific Research Center, Azerbaijan State Economic University (UNEC), H. Aliyev str. 135, Az 1063, Baku, Azerbaijan; c Department of Aircraft Electrics and Electronics, School of Applied Sciences, Cappadocia University, Mustafapaşa, 50420 Ürgüp, Nevşehir, Turkey; d Department of Physics, Faculty of Sciences, Erciyes University, 38039 Kayseri, Turkey; e Acad. Sci. Republ. Tadzhikistan, Kh. Yu. Yusufbekov Pamir Biol. Inst., 1 Kholdorova St, Khorog 736002, Gbao, Tajikistan

**Keywords:** crystal structure, imidazolidine ring, pyridine ring, hydrogen bond, Hirshfeld surface analysis

## Abstract

In the title compound, C_22_H_17_N_5_·CH_4_O, the imidazolidine ring of the 1,2,3,5,6,7-hexa­hydro­imidazo[1,2-*a*]pyridine ring system is a twisted envelope, while the 1,2,3,4-tetra­hydro­pyridine ring adopts a twisted boat conformation. In the crystal, pairs of mol­ecules are linked by O—H⋯N and N—H⋯O hydrogen bonds *via* two methanol mol­ecules, forming a centrosymmetric *R* ^4^_4_(16) ring motif. These motifs are connected to each other by C—H⋯N hydrogen bonds and form columns along the *a* axis. The columns form a stable mol­ecular packing, being connected to each other by van der Waals inter­actions. A Hirshfeld surface analysis indicates that the most significant contributions to the crystal packing are from H⋯H (43.8%), N⋯H/H⋯N (31.7%) and C⋯H/H⋯C (18.4%) contacts.

## Abstract 2

In the crystal, O—H⋯N and N—H⋯O hydrogen bonds link pairs of mol­ecules *via* two methanol mol­ecules. These mol­ecules are connected to each other by C—H⋯N hydrogen bonds and form columns along the *a*-axis direction.

## Chemical context   

**Figure 1:**
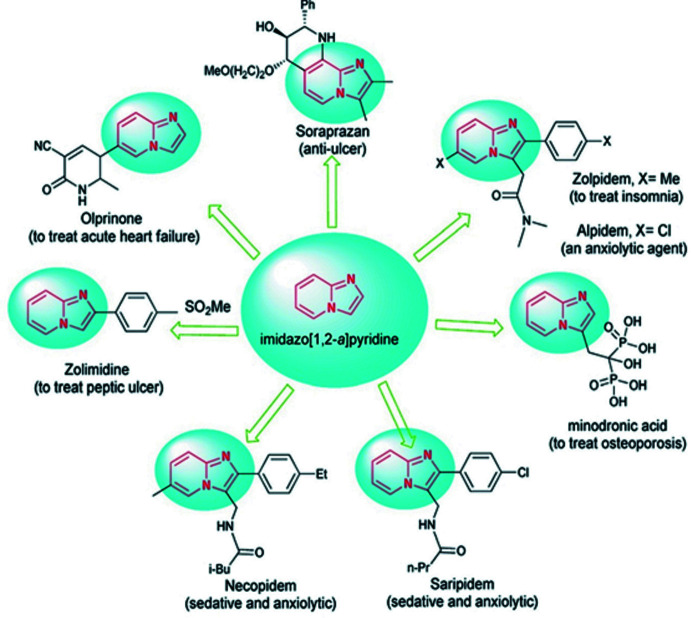
Drugs containing the imidazo[1,2-*a*]pyridine motif.

Having a great methodological diversity, C—C and C—*X* (where *X* is a heteroatom) bond-forming reactions lie at the heart of synthetic organic chemistry (Khalilov *et al.*, 2018**a* ▸,b* ▸; Maharramov *et al.*, 2019 ▸; Cheng & Mankad, 2020 ▸). They allow the construction of complex mol­ecular structures and the introduction of various substituents. Nowadays, researchers are constantly trying to develop new methods in these directions for the syntheses of structurally diverse valuable mol­ecular entities. These approaches have successfully found application in the building of carbo- and heterocyclic ring systems (Naghiyev *et al.*, 2020 ▸; Mamedov *et al.*, 2019 ▸). In heterocyclic ring systems, the use of nitro­gen as a bridgehead atom is being assessed widely. Bridgehead nitro­gen heterocycles incorporating an imidazole ring are widespread structural motifs in a diverse range of compounds having application in medicinal chemistry, coordination chemistry, catalysis and materials science (Asadov *et al.*, 2016 ▸; Ma *et al.*, 2017*a* ▸,*b* ▸, 2020 ▸, 2021 ▸; Maharramov *et al.*, 2010 ▸, 2018 ▸; Mahmoudi *et al.*, 2017 ▸, 2019 ▸; Mahmudov *et al.*, 2019 ▸, 2020 ▸). Various synthetic drugs, such as soraprazan, alpidem, olprinone, saripidem, necopidem, minodronic acid, zolimidine and zolpidem containing the imidazo[1,2-*a*]pyridine moiety (Fig. 1 ▸) have already been used in medical practice (Hosseini & Bayat, 2018 ▸).

In the framework of our ongoing structural studies (Naghiyev *et al.*, 2021 ▸), herein we report the crystal structure and Hirshfeld surface analysis of the title compound, 5,7-diphenyl-1,2,3,5,6,7-hexa­hydro­imidazo[1,2-*a*]pyridine-6,6,8-tricarbo­nitrile methanol monosolvate.
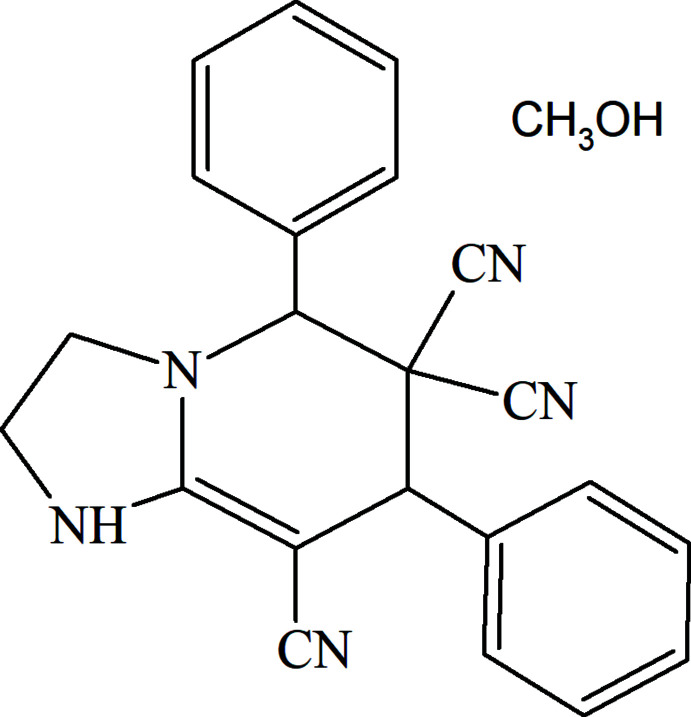


## Structural commentary   

**Figure 2:**
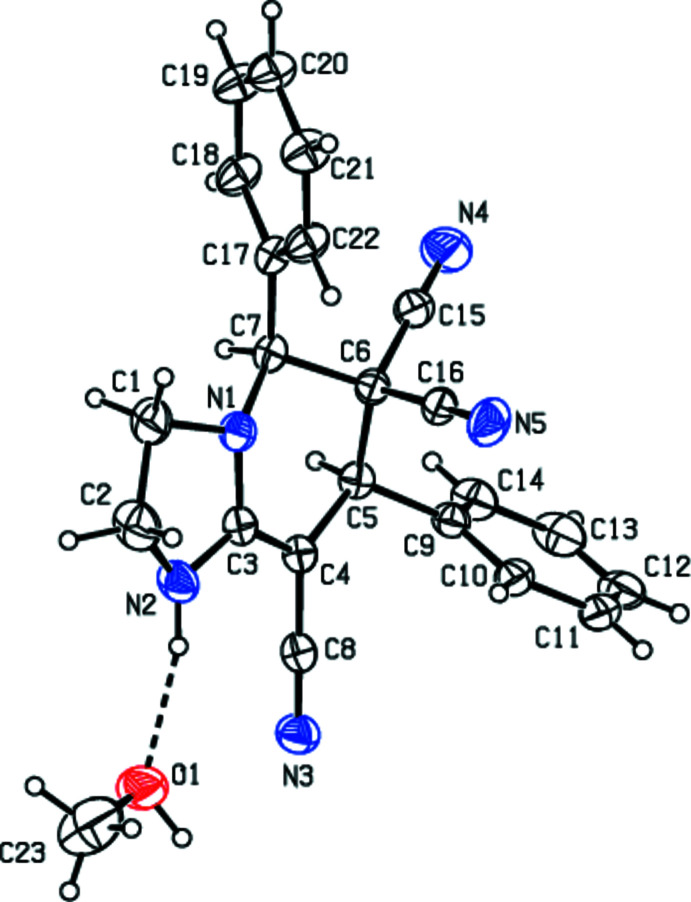
The mol­ecular structure of the title compound. Displacement ellipsoids are drawn at the 30% probability level. The dashed line indicates a N—H⋯O hydrogen bond.

In the title compound, (Fig. 2 ▸), the imidazolidine ring (N1/N2/C1–C3) of the 1,2,3,5,6,7-hexa­hydro­imidazo[1,2-*a*]pyridine ring system (N1/N2/C1–C7) is a twisted envelope [with puckering parameters (Cremer & Pople, 1975 ▸) *Q*(2) = 0.2844 (16) Å and φ(2) = 226.3 (3)°], while the 1,2,3,4-tetra­hydro­pyridine ring (N1/C3–C7) adopts a twisted boat conformation with *Q*_T_ = 0.5368 (14) Å, θ = 135.38 (15)°, φ = 82.6 (2)°. The C9–C14 and C17–C22 phenyl rings, which are attached to C5 and C7, respectively, are in equatorial positions (Fig. 2 ▸) and make dihedral angles of 64.00 (7) and 65.90 (7)°, respectively, with the mean plane of the 1,2,3,5,6,7-hexa­hydro­imidazo[1,2-*a*]pyridine ring system. The dihedral angle between the phenyl rings is 61.43 (8)°. The mol­ecular conformation of the title compound is stabilized by an N2—H2*N*⋯O1 hydrogen bond (Fig. 2 ▸, Table 1 ▸).

## Supra­molecular features and Hirshfeld surface analysis   

**Figure 3:**
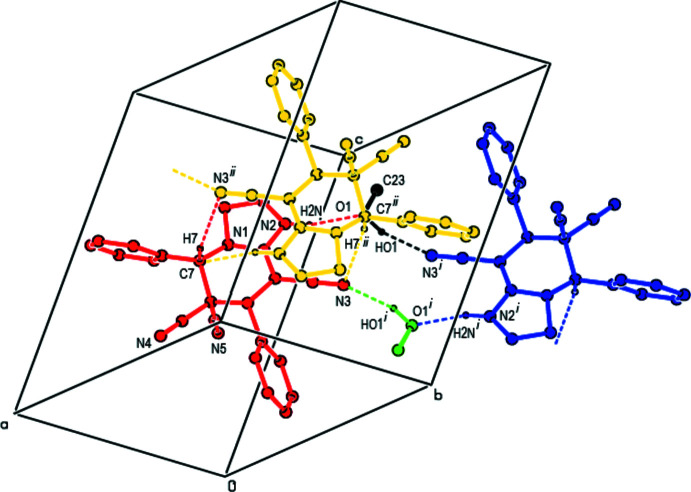
Details of the O—H⋯N, C—H⋯N and N—H⋯O hydrogen bonds (dashed lines) in the unit cell of the title compound. H atoms not involved in hydrogen bonding have been omitted for clarity. [Symmetry codes: (i) −*x*, −*y* + 1, −*z* + 1; (ii) −*x* + 1, −*y* + 1, −*z* + 1.]

**Figure 4:**
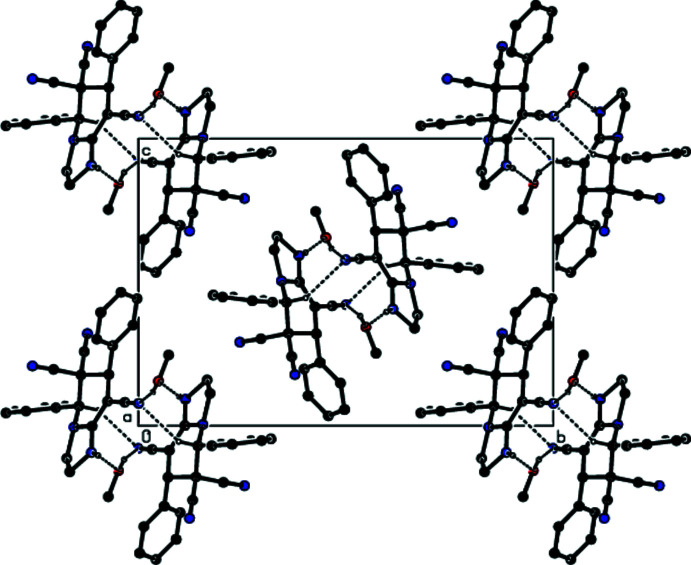
A view along the *a* axis of the columns that are formed by the O—H⋯N, C—H⋯N and N—H⋯O hydrogen bonds (dashed lines) in the title compound. H atoms not involved in hydrogen bonding have been omitted for clarity.

In the crystal, pairs of mol­ecules are linked by O—H⋯N, C—H⋯N and N—H⋯O hydrogen bonds *via* two methanol mol­ecules, forming a centrosymmetric 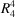
(16) ring motif (Bernstein *et al.*, 1995 ▸; Table 1 ▸; Fig. 3 ▸). These motifs are connected to each other by C—H⋯N hydrogen bonds and form columns along the *a*-axis direction (Figs. 4 ▸ and 5 ▸). The columns form a stable mol­ecular packing, being connected to each other by van der Waals inter­actions.

**Figure 6:**
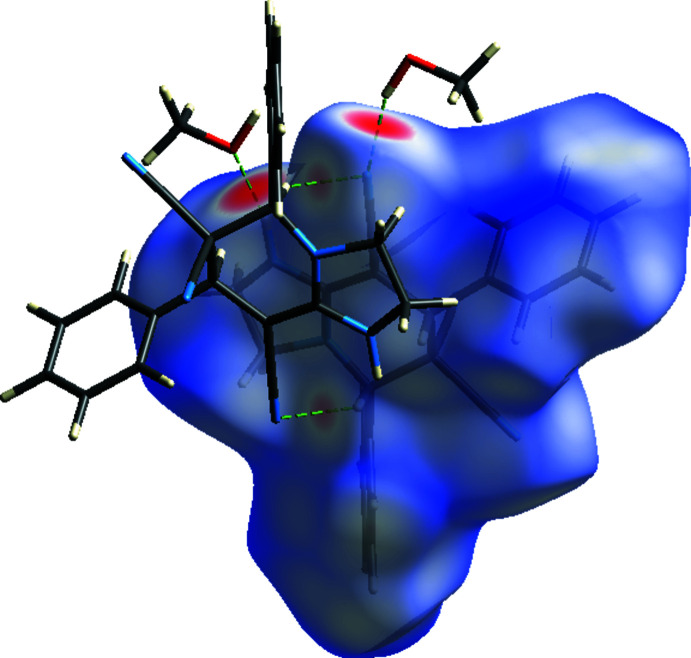
The three-dimensional Hirshfeld surface of the title compound plotted over *d*_norm_ in the range −0.5585 to +1.5646 a.u. The O—H⋯N, C—H⋯O and N—H⋯O hydrogen bonds are shown as dashed lines.

To further investigate and visualize the inter­molecular inter­actions of the title compound, the *CrystalExplorer* program (Turner *et al.*, 2017 ▸) was used. The inter­actions between the corresponding donor and acceptor atoms are visualized as bright-red spots on the Hirshfeld surface mapped over *d*_norm_ (Fig. 6 ▸), corresponding to O1—H*O*1⋯N3, C7—H7⋯N3 and N2—H2*N*⋯O1 hydrogen bonds. The other red spots correspond to weaker van der Waals inter­actions, of which the details are listed in Table 2 ▸.

**Figure 7:**
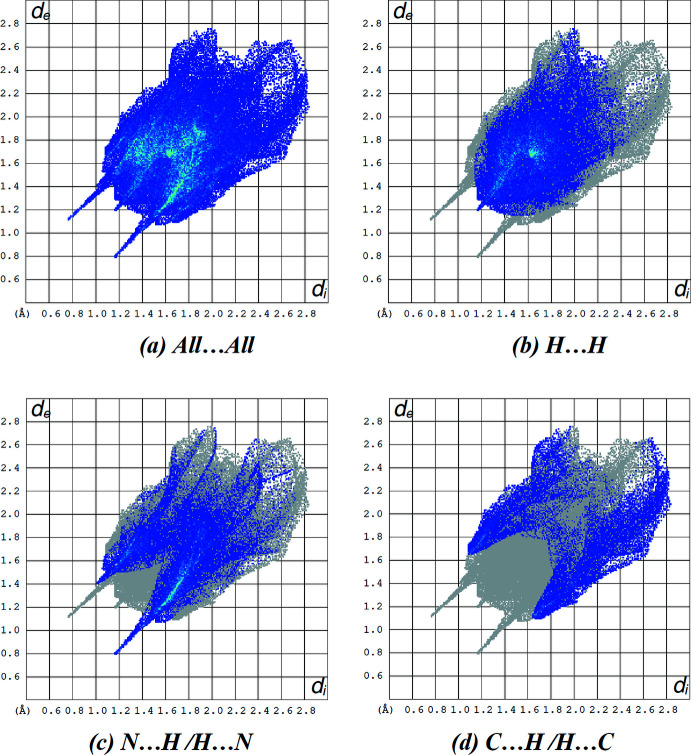
The two-dimensional fingerprint plots of the title compound, showing (*a*) all inter­actions, and delineated into (*b*) H⋯H, (*c*) N⋯H/H⋯N, and (*d*) C⋯H/H⋯C inter­actions [*d*_e_ and *d*_i_ represent the distances from a point on the Hirshfeld surface to the nearest atoms outside (external) and inside (inter­nal) the surface, respectively].

The overall two-dimensional fingerprint plot of the title structure and H⋯H, N⋯H/H⋯N and C⋯H/H⋯C contacts are illustrated in Fig. 7 ▸ *a*–*d*). The greatest contribution to the overall Hirshfeld surface results from H⋯H contacts with a 43.8% contribution (Fig. 7 ▸ *b*). The relative contributions of the other inter­actions in descending order are: N⋯H/H⋯N (31.7%), C⋯H/H⋯C (18.4%), O⋯H/H⋯O (2.6%), C⋯C (2.4%), N⋯O/O⋯N (0.1%) and C⋯O/O⋯C (0.1%). The large contributions of H⋯H, N⋯H/H⋯N and C⋯H/H⋯C inter­actions suggest that van der Waals inter­actions and hydrogen bonding play the major roles in the crystal packing (Hathwar *et al.*, 2015 ▸).

## Database survey   

A survey of the Cambridge Structural Database (CSD version 5.41, update of March 2020; Groom *et al.*, 2016 ▸) reveals two related compounds having the 1,2,3,5,6,7-hexa­hydro­imidazo[1,2-*a*]pyridine ring system of the title compound: ethyl 8-benzoyl-5-oxo-7-phenyl-1,2,3,5,6,7-hexa­hydro­imid­azo[1,2-*a*]pyridine-6-carboxyl­ate (refcode ADETUZ; Yu *et al.*, 2006 ▸) and 1-[(6-chloro­pyridin-3-yl)meth­yl]-5-eth­oxy-8-nitro-1,2,3,5,6,7-hexa­hydro­imidazo[1,2-*a*]pyridine (BUDZAC; Tian *et al.*, 2009 ▸).

In ADETUZ, the six-membered ring adopts a twist-boat conformation. The mol­ecules form dimeric associations *via* inversion-generated pairs of N—H⋯O hydrogen bonds. In BUDZAC, the fused pyridine ring adopts a twisted sofa conformation. The mol­ecular structure features close intra­molecular C—H⋯N and C—H⋯O hydrogen bonding.

## Synthesis and crystallization   

To a solution of benzyl­idenemalono­nitrile (0.78 g; 5.1 mmol) in ethanol (30 mL), ethyl­enedi­amine (0.31 g; 5.2 mmol) was added and the mixture was refluxed for 7 h. Then 25 mL of ethanol were removed from the reaction mixture, which was left overnight. The precipitated crystals were separated by filtration and recrystallized from methanol (yield 47%; m.p. 443–444 K).

^1^H NMR (300 MHz, DMSO-*d*_6_): 3.18 (*t*, 2H, NCH_2_); 3.42 (*t*, 2H, NCH_2_); 4.79 (*s*, 1H, CH-Ar); 5.19 (*s*, 1H, CH-Ar); 7.41–7.58 (*m*, 10H, 10Ar-H); 7.70 (*s*, 1H, NH).^13^C NMR (75 MHz, DMSO-*d*_6_): 42.04 (NCH_2_), 47.67 (CH-Ar), 48.41 (C_quart_), 48.64 (=C_quart_), 49.05 (NCH_2_), 63.46 (CH-Ar), 112.91 (CN), 113.69 (CN), 121.24 (CN), 129.03 (4CH_arom_), 129.42 (2CH_arom_), 129.72 (CH_arom_), 129.96 (2CH_arom_), 130.85 (CH_arom_), 133.25 (C_arom_),135.90 (C_ar._), 162.08 (=C_quart_).

## Refinement   

Crystal data, data collection and structure refinement details are summarized in Table 3 ▸. The C-bound H atoms were placed in calculated positions (C—H = 0.93–0.98 Å) and refined as riding with *U*_iso_(H) = 1.2 or 1.5*U*_eq_(C). The H atoms of the amine and hydroxyl groups were located in a difference map [N2—H2*N* = 0.887 (18) Å and O1—H*O*1 = 0.92 (3) Å] and were refined with the constraint *U*_iso_(H) = 1.2*U*_eq_(N) or 1.5*U*_eq_(O).

## Supplementary Material

Crystal structure: contains datablock(s) global, I. DOI: 10.1107/S2056989021004655/vm2248sup1.cif

Structure factors: contains datablock(s) I. DOI: 10.1107/S2056989021004655/vm2248Isup2.hkl

Supporting information file. DOI: 10.1107/S2056989021004655/vm2248Isup3.cml

CCDC reference: 2081515

Additional supporting information:  crystallographic information; 3D view; checkCIF report

## Figures and Tables

**Figure 5:**
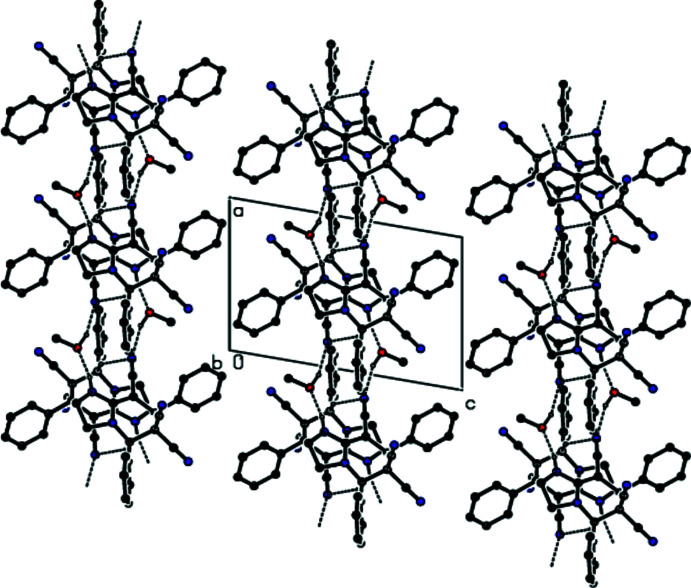
A part of view along the *b* axis of the columns shown in Fig. 4 ▸. H atoms not involved in hydrogen bonding have been omitted for clarity. Dashed lines indicate hydrogen bonds.

**Table 1:** Hydrogen-bond geometry (Å, °)

*D*—H⋯*A*	*D*—H	H⋯*A*	*D*⋯*A*	*D*—H⋯*A*
O1—H*O*1⋯N3^i^	0.92 (3)	2.02 (3)	2.907 (2)	161 (2)
N2—H2*N*⋯O1	0.887 (18)	2.004 (18)	2.870 (2)	165.0 (16)
C7—H7⋯N3^ii^	0.98	2.52	3.3742 (19)	146

Symmetry codes: (i) -x, -y+1, -z+1; (ii) -x+1, -y+1, -z+1.

**Table 2:** Summary of short inter­atomic contacts (Å) in the title compound

Contact	Distance	Symmetry operation
H2*N*⋯O1	2.00	*x*, *y*, *z*
H7⋯N3	2.52	1 − *x*, 1 − *y*, 1 − *z*
N3⋯H18	2.75	−1 + *x*, *y*, *z*
N3⋯H*O*1	2.02	−*x*, 1 − *y*, 1 − *z*
N5⋯H1*B*	2.75	−{1\over 2} + *x*, {1\over 2} − *y*, −{1\over 2} + *z*
N5⋯H23*B*	2.77	{1\over 2} + *x*, {1\over 2} − *y*, −{1\over 2} + *z*
H12⋯H12	2.54	−*x*, 1 − *y*, −*z*
C20⋯H2*B*	3.02	{1\over 2} + *x*, {1\over 2} − *y*, −{1\over 2} + *z*
H5⋯O1	2.79	1 − *x*, 1 − *y*, 1 − *z*
H12⋯C23	3.07	*x*, *y*, −1 + *z*

**Table 3:** Experimental details

Crystal data	
Chemical formula	C_22_H_17_N_5_·CH_4_O
*M*_r_	383.45
Crystal system, space group	Monoclinic, *P*2_1_/*n*
Temperature (K)	296
*a*, *b*, *c* (Å)	8.5517 (10), 18.781 (2), 13.1925 (14)
β (°)	99.757 (4)
*V* (Å^3^)	2088.2 (4)
*Z*	4
Radiation type	Mo *K*α
μ (mm^−1^)	0.08
Crystal size (mm)	0.28 × 0.26 × 0.25

Data collection	
Diffractometer	Bruker APEXII CCD
No. of measured, independent and observed [*I* > 2σ(*I*)] reflections	33652, 4239, 3656
*R*_int_	0.033
(sin θ/λ)_max_ (Å^−1^)	0.626

Refinement	
*R*[*F* ^2^ > 2σ(*F* ^2^)], *wR*(*F* ^2^), *S*	0.046, 0.123, 1.03
No. of reflections	4239
No. of parameters	269
H-atom treatment	H atoms treated by a mixture of independent and constrained refinement
Δρ_max_, Δρ_min_ (e Å^−3^)	0.31, −0.23

Computer programs: *APEX2* (Bruker, 2018 ▸), *SAINT* (Bruker, 2013 ▸), *SHELXT2014/5* (Sheldrick, 2015*a* ▸), *SHELXL2018/3* (Sheldrick, 2015*b* ▸), *ORTEP-3 for Windows* (Farrugia, 2012 ▸) and *PLATON* (Spek, 2020 ▸).

## supplementary crystallographic information

### Crystal data

**Table:**

C_22_H_17_N_5_·CH_4_O	*F*(000) = 808
*M**_r_* = 383.45	*D*_x_ = 1.220 Mg m^−^^3^
Monoclinic, *P*2_1_/*n*	Mo *K*α radiation, λ = 0.71073 Å
Hall symbol: -P 2yn	Cell parameters from 9885 reflections
*a* = 8.5517 (10) Å	θ = 3.1–26.4°
*b* = 18.781 (2) Å	µ = 0.08 mm^−^^1^
*c* = 13.1925 (14) Å	*T* = 296 K
β = 99.757 (4)°	Block, colourless
*V* = 2088.2 (4) Å^3^	0.28 × 0.26 × 0.25 mm
*Z* = 4	

### Data collection

**Table:**

Bruker APEXII CCD diffractometer	*R*_int_ = 0.033
φ and ω scans	θ_max_ = 26.4°, θ_min_ = 2.7°
33652 measured reflections	*h* = −10→10
4239 independent reflections	*k* = −23→23
3656 reflections with *I* > 2σ(*I*)	*l* = −16→16

### Refinement

**Table:**

Refinement on *F*^2^	0 restraints
Least-squares matrix: full	Hydrogen site location: mixed
*R*[*F*^2^ > 2σ(*F*^2^)] = 0.046	H atoms treated by a mixture of independent and constrained refinement
*wR*(*F*^2^) = 0.123	*w* = 1/[σ^2^(*F*_o_^2^) + (0.0553*P*)^2^ + 0.5899*P*] where *P* = (*F*_o_^2^ + 2*F*_c_^2^)/3
*S* = 1.02	(Δ/σ)_max_ < 0.001
4239 reflections	Δρ_max_ = 0.31 e Å^−^^3^
269 parameters	Δρ_min_ = −0.23 e Å^−^^3^

### Special details

**Table:**

Geometry. Bond distances, angles etc. have been calculated using the rounded fractional coordinates. All su's are estimated from the variances of the (full) variance-covariance matrix. The cell esds are taken into account in the estimation of distances, angles and torsion angles
Refinement. Refinement on F^2^ for ALL reflections except those flagged by the user for potential systematic errors. Weighted R-factors wR and all goodnesses of fit S are based on F^2^, conventional R-factors R are based on F, with F set to zero for negative F^2^. The observed criterion of F^2^ > 2sigma(F^2^) is used only for calculating -R-factor-obs etc. and is not relevant to the choice of reflections for refinement. R-factors based on F^2^ are statistically about twice as large as those based on F, and R-factors based on ALL data will be even larger.

### Fractional atomic coordinates and isotropic or equivalent isotropic displacement parameters (Å^2^)

**Table:**

	*x*	*y*	*z*	*U*_iso_*/*U*_eq_	
N1	0.60712 (14)	0.34477 (6)	0.50461 (8)	0.0455 (3)	
N2	0.42807 (17)	0.38674 (7)	0.59278 (9)	0.0554 (4)	
N3	0.16518 (16)	0.50048 (7)	0.42172 (10)	0.0564 (4)	
N4	0.77545 (19)	0.37704 (11)	0.17841 (12)	0.0818 (7)	
N5	0.43619 (17)	0.24442 (8)	0.29156 (13)	0.0687 (5)	
C1	0.6680 (2)	0.32722 (10)	0.61313 (11)	0.0610 (5)	
O1	0.15621 (16)	0.45171 (9)	0.65565 (10)	0.0796 (5)	
C2	0.5187 (2)	0.33436 (11)	0.66018 (13)	0.0728 (6)	
C3	0.47744 (16)	0.38817 (7)	0.50125 (10)	0.0416 (4)	
C4	0.41241 (15)	0.42550 (7)	0.41462 (9)	0.0400 (4)	
C5	0.49374 (15)	0.42867 (7)	0.32123 (9)	0.0400 (3)	
C6	0.60291 (14)	0.36144 (7)	0.32248 (9)	0.0396 (4)	
C7	0.71275 (15)	0.35675 (7)	0.43062 (10)	0.0406 (3)	
C8	0.27705 (16)	0.46706 (7)	0.41791 (10)	0.0430 (4)	
C9	0.38002 (16)	0.43787 (7)	0.22050 (10)	0.0435 (4)	
C10	0.24007 (17)	0.39947 (9)	0.19806 (11)	0.0529 (5)	
C11	0.14001 (19)	0.40923 (12)	0.10485 (13)	0.0698 (6)	
C12	0.1790 (2)	0.45789 (13)	0.03484 (12)	0.0768 (7)	
C13	0.3160 (3)	0.49685 (11)	0.05680 (13)	0.0726 (7)	
C14	0.4164 (2)	0.48700 (9)	0.14907 (12)	0.0567 (5)	
C15	0.70149 (17)	0.36934 (9)	0.24146 (11)	0.0519 (4)	
C16	0.50897 (16)	0.29533 (7)	0.30346 (11)	0.0456 (4)	
C17	0.84126 (15)	0.30107 (7)	0.43683 (10)	0.0422 (4)	
C18	0.99408 (17)	0.32313 (8)	0.43065 (13)	0.0555 (5)	
C19	1.11587 (18)	0.27445 (10)	0.43609 (15)	0.0650 (6)	
C20	1.08799 (19)	0.20309 (9)	0.44872 (14)	0.0622 (6)	
C21	0.93680 (19)	0.18065 (8)	0.45511 (14)	0.0615 (5)	
C22	0.81374 (17)	0.22886 (8)	0.44866 (12)	0.0519 (4)	
C23	0.1184 (3)	0.4273 (2)	0.74675 (18)	0.1210 (12)	
H1A	0.74920	0.36056	0.64351	0.0730*	
H1B	0.71018	0.27920	0.62033	0.0730*	
H2A	0.46245	0.28943	0.65878	0.0870*	
H2B	0.54270	0.35144	0.73049	0.0870*	
H2N	0.334 (2)	0.4024 (10)	0.6028 (14)	0.0660*	
H5	0.56337	0.47045	0.32956	0.0480*	
H7	0.76386	0.40317	0.44550	0.0490*	
H10	0.21323	0.36709	0.24562	0.0640*	
H11	0.04673	0.38302	0.08952	0.0840*	
H12	0.11175	0.46428	−0.02770	0.0920*	
H13	0.34110	0.52988	0.00955	0.0870*	
H14	0.50947	0.51345	0.16372	0.0680*	
H18	1.01463	0.37126	0.42275	0.0670*	
H19	1.21761	0.28995	0.43119	0.0780*	
H20	1.17047	0.17038	0.45289	0.0750*	
H21	0.91735	0.13251	0.46387	0.0740*	
H22	0.71184	0.21295	0.45224	0.0620*	
HO1	0.061 (3)	0.4657 (15)	0.617 (2)	0.1190*	
H23A	0.21254	0.42549	0.79796	0.1810*	
H23B	0.07336	0.38048	0.73674	0.1810*	
H23C	0.04282	0.45890	0.76915	0.1810*	

### Atomic displacement parameters (Å^2^)

**Table:**

	*U*^11^	*U*^22^	*U*^33^	*U*^12^	*U*^13^	*U*^23^
N1	0.0484 (6)	0.0509 (6)	0.0359 (6)	0.0044 (5)	0.0037 (5)	0.0042 (5)
N2	0.0646 (8)	0.0647 (8)	0.0388 (6)	0.0088 (6)	0.0142 (6)	0.0043 (5)
N3	0.0557 (7)	0.0551 (7)	0.0591 (8)	0.0089 (6)	0.0120 (6)	−0.0015 (6)
N4	0.0656 (9)	0.1250 (15)	0.0605 (9)	0.0014 (9)	0.0267 (8)	0.0042 (9)
N5	0.0602 (8)	0.0552 (8)	0.0845 (10)	−0.0080 (7)	−0.0053 (7)	−0.0160 (7)
C1	0.0707 (10)	0.0703 (10)	0.0390 (7)	0.0103 (8)	0.0004 (7)	0.0099 (7)
O1	0.0639 (7)	0.1204 (12)	0.0577 (7)	0.0139 (8)	0.0191 (6)	0.0105 (7)
C2	0.0933 (13)	0.0838 (12)	0.0428 (8)	0.0176 (10)	0.0161 (8)	0.0159 (8)
C3	0.0469 (7)	0.0407 (6)	0.0369 (6)	−0.0037 (5)	0.0067 (5)	−0.0032 (5)
C4	0.0432 (7)	0.0391 (6)	0.0373 (6)	−0.0009 (5)	0.0057 (5)	−0.0018 (5)
C5	0.0414 (6)	0.0390 (6)	0.0396 (6)	−0.0032 (5)	0.0067 (5)	0.0017 (5)
C6	0.0377 (6)	0.0438 (7)	0.0370 (6)	−0.0019 (5)	0.0051 (5)	−0.0007 (5)
C7	0.0397 (6)	0.0399 (6)	0.0400 (6)	−0.0044 (5)	0.0009 (5)	−0.0008 (5)
C8	0.0499 (7)	0.0397 (7)	0.0393 (6)	−0.0026 (6)	0.0076 (5)	−0.0017 (5)
C9	0.0451 (7)	0.0480 (7)	0.0381 (6)	0.0099 (6)	0.0095 (5)	0.0036 (5)
C10	0.0431 (7)	0.0721 (10)	0.0434 (7)	0.0063 (7)	0.0067 (6)	0.0015 (7)
C11	0.0436 (8)	0.1130 (15)	0.0509 (9)	0.0185 (9)	0.0027 (7)	−0.0113 (9)
C12	0.0699 (11)	0.1208 (16)	0.0386 (8)	0.0531 (12)	0.0064 (7)	0.0081 (9)
C13	0.0874 (13)	0.0836 (12)	0.0502 (9)	0.0394 (11)	0.0218 (9)	0.0238 (8)
C14	0.0666 (9)	0.0564 (9)	0.0501 (8)	0.0135 (7)	0.0182 (7)	0.0125 (7)
C15	0.0445 (7)	0.0657 (9)	0.0455 (7)	0.0031 (6)	0.0076 (6)	0.0017 (6)
C16	0.0407 (7)	0.0467 (7)	0.0466 (7)	0.0017 (6)	−0.0004 (5)	−0.0075 (6)
C17	0.0387 (6)	0.0444 (7)	0.0412 (7)	−0.0035 (5)	−0.0002 (5)	−0.0002 (5)
C18	0.0436 (7)	0.0490 (8)	0.0716 (10)	−0.0085 (6)	0.0034 (7)	0.0044 (7)
C19	0.0385 (7)	0.0712 (11)	0.0841 (12)	−0.0037 (7)	0.0074 (7)	0.0060 (9)
C20	0.0488 (8)	0.0629 (10)	0.0731 (11)	0.0106 (7)	0.0052 (7)	0.0055 (8)
C21	0.0582 (9)	0.0453 (8)	0.0798 (11)	0.0022 (7)	0.0080 (8)	0.0084 (7)
C22	0.0418 (7)	0.0462 (7)	0.0663 (9)	−0.0052 (6)	0.0053 (6)	0.0051 (6)
C23	0.0825 (15)	0.205 (3)	0.0758 (14)	−0.0012 (18)	0.0142 (12)	0.0563 (18)

### Geometric parameters (Å, º)

**Table:**

N1—C1	1.4754 (18)	C17—C22	1.390 (2)
N1—C3	1.3710 (18)	C17—C18	1.387 (2)
N1—C7	1.4554 (17)	C18—C19	1.379 (2)
N2—C2	1.458 (2)	C19—C20	1.376 (3)
N2—C3	1.3453 (18)	C20—C21	1.376 (2)
N3—C8	1.1524 (19)	C21—C22	1.380 (2)
N4—C15	1.136 (2)	C1—H1A	0.9700
N5—C16	1.137 (2)	O1—HO1	0.92 (3)
C1—C2	1.517 (2)	C1—H1B	0.9700
O1—C23	1.375 (3)	C2—H2A	0.9700
N2—H2N	0.887 (18)	C2—H2B	0.9700
C3—C4	1.3753 (18)	C5—H5	0.9800
C4—C8	1.4029 (19)	C7—H7	0.9800
C4—C5	1.5151 (17)	C10—H10	0.9300
C5—C6	1.5687 (18)	C11—H11	0.9300
C5—C9	1.5177 (18)	C12—H12	0.9300
C6—C7	1.5720 (18)	C13—H13	0.9300
C6—C16	1.4770 (19)	C14—H14	0.9300
C6—C15	1.4767 (19)	C18—H18	0.9300
C7—C17	1.5091 (19)	C19—H19	0.9300
C9—C14	1.391 (2)	C20—H20	0.9300
C9—C10	1.386 (2)	C21—H21	0.9300
C10—C11	1.386 (2)	C22—H22	0.9300
C11—C12	1.380 (3)	C23—H23A	0.9600
C12—C13	1.370 (3)	C23—H23B	0.9600
C13—C14	1.378 (3)	C23—H23C	0.9600
			
C1—N1—C3	108.34 (11)	C17—C22—C21	120.35 (14)
C1—N1—C7	121.89 (12)	N1—C1—H1A	112.00
C3—N1—C7	118.49 (11)	N1—C1—H1B	112.00
C2—N2—C3	110.22 (13)	C2—C1—H1A	112.00
N1—C1—C2	101.20 (13)	C2—C1—H1B	112.00
N2—C2—C1	101.97 (14)	H1A—C1—H1B	109.00
C3—N2—H2N	123.9 (12)	C23—O1—HO1	105.2 (17)
C2—N2—H2N	122.3 (12)	N2—C2—H2B	111.00
N1—C3—C4	122.75 (12)	C1—C2—H2A	111.00
N2—C3—C4	127.72 (13)	C1—C2—H2B	111.00
N1—C3—N2	109.53 (12)	H2A—C2—H2B	109.00
C5—C4—C8	119.88 (11)	N2—C2—H2A	111.00
C3—C4—C5	121.27 (12)	C4—C5—H5	107.00
C3—C4—C8	118.52 (12)	C9—C5—H5	107.00
C6—C5—C9	113.12 (10)	C6—C5—H5	107.00
C4—C5—C9	113.71 (11)	N1—C7—H7	108.00
C4—C5—C6	108.31 (10)	C17—C7—H7	108.00
C5—C6—C15	108.86 (11)	C6—C7—H7	108.00
C5—C6—C16	111.67 (10)	C9—C10—H10	120.00
C7—C6—C16	109.34 (11)	C11—C10—H10	120.00
C15—C6—C16	108.80 (11)	C12—C11—H11	120.00
C7—C6—C15	109.63 (11)	C10—C11—H11	120.00
C5—C6—C7	108.52 (10)	C11—C12—H12	120.00
C6—C7—C17	113.94 (11)	C13—C12—H12	120.00
N1—C7—C6	105.90 (10)	C14—C13—H13	120.00
N1—C7—C17	112.68 (11)	C12—C13—H13	120.00
N3—C8—C4	178.94 (14)	C9—C14—H14	120.00
C10—C9—C14	118.99 (13)	C13—C14—H14	120.00
C5—C9—C10	122.00 (12)	C19—C18—H18	120.00
C5—C9—C14	119.01 (13)	C17—C18—H18	120.00
C9—C10—C11	120.11 (15)	C18—C19—H19	120.00
C10—C11—C12	119.91 (16)	C20—C19—H19	120.00
C11—C12—C13	120.48 (16)	C21—C20—H20	120.00
C12—C13—C14	119.84 (18)	C19—C20—H20	120.00
C9—C14—C13	120.66 (17)	C20—C21—H21	120.00
N4—C15—C6	178.24 (18)	C22—C21—H21	120.00
N5—C16—C6	178.13 (16)	C17—C22—H22	120.00
C7—C17—C18	118.34 (12)	C21—C22—H22	120.00
C18—C17—C22	118.52 (13)	O1—C23—H23A	109.00
C7—C17—C22	123.14 (12)	O1—C23—H23B	109.00
C17—C18—C19	120.67 (14)	O1—C23—H23C	109.00
C18—C19—C20	120.48 (15)	H23A—C23—H23B	109.00
C19—C20—C21	119.31 (15)	H23A—C23—H23C	109.00
C20—C21—C22	120.66 (14)	H23B—C23—H23C	109.00
			
C3—N1—C1—C2	26.59 (16)	C9—C5—C6—C7	−179.03 (10)
C7—N1—C1—C2	169.55 (13)	C9—C5—C6—C15	61.71 (14)
C7—N1—C3—N2	−158.46 (12)	C4—C5—C6—C15	−171.28 (11)
C1—N1—C7—C6	171.10 (12)	C16—C6—C7—N1	−57.45 (13)
C1—N1—C3—N2	−14.05 (16)	C16—C6—C7—C17	66.96 (14)
C3—N1—C7—C17	−174.68 (11)	C15—C6—C7—C17	−52.24 (15)
C1—N1—C3—C4	166.57 (13)	C5—C6—C7—N1	64.58 (12)
C7—N1—C3—C4	22.16 (19)	C5—C6—C7—C17	−171.02 (10)
C1—N1—C7—C17	45.91 (17)	C15—C6—C7—N1	−176.64 (11)
C3—N1—C7—C6	−49.49 (14)	N1—C7—C17—C18	−137.73 (13)
C3—N2—C2—C1	22.03 (17)	N1—C7—C17—C22	42.12 (18)
C2—N2—C3—C4	173.60 (14)	C6—C7—C17—C18	101.58 (15)
C2—N2—C3—N1	−5.74 (17)	C6—C7—C17—C22	−78.57 (16)
N1—C1—C2—N2	−28.10 (16)	C5—C9—C10—C11	−179.63 (15)
N1—C3—C4—C5	−8.3 (2)	C14—C9—C10—C11	1.2 (2)
N1—C3—C4—C8	178.32 (12)	C5—C9—C14—C13	−179.97 (16)
N2—C3—C4—C5	172.44 (13)	C10—C9—C14—C13	−0.8 (2)
N2—C3—C4—C8	−0.9 (2)	C9—C10—C11—C12	−0.8 (3)
C3—C4—C5—C6	24.53 (16)	C10—C11—C12—C13	−0.1 (3)
C8—C4—C5—C6	−162.18 (11)	C11—C12—C13—C14	0.6 (3)
C8—C4—C5—C9	−35.51 (17)	C12—C13—C14—C9	−0.1 (3)
C3—C4—C5—C9	151.19 (12)	C7—C17—C18—C19	179.89 (15)
C4—C5—C6—C7	−52.02 (13)	C22—C17—C18—C19	0.0 (2)
C9—C5—C6—C16	−58.43 (14)	C7—C17—C22—C21	−179.19 (14)
C4—C5—C9—C10	−43.99 (18)	C18—C17—C22—C21	0.7 (2)
C4—C5—C9—C14	135.18 (14)	C17—C18—C19—C20	−0.6 (3)
C6—C5—C9—C10	80.12 (16)	C18—C19—C20—C21	0.5 (3)
C6—C5—C9—C14	−100.71 (15)	C19—C20—C21—C22	0.2 (3)
C4—C5—C6—C16	68.57 (13)	C20—C21—C22—C17	−0.8 (3)

### Hydrogen-bond geometry (Å, º)

**Table:**

*D*—H···*A*	*D*—H	H···*A*	*D*···*A*	*D*—H···*A*
O1—H*O*1···N3^i^	0.92 (3)	2.02 (3)	2.907 (2)	161 (2)
N2—H2*N*···O1	0.887 (18)	2.004 (18)	2.870 (2)	165.0 (16)
C7—H7···N3^ii^	0.98	2.52	3.3742 (19)	146

Symmetry codes: (i) −*x*, −*y*+1, −*z*+1; (ii) −*x*+1, −*y*+1, −*z*+1.
